# Diseases Admitted under the Care of the Physicians of the Westminster Hospital, from the 20th of May to the 21st of June, 1800

**Published:** 1800-07

**Authors:** 


					*4 State of Difeajes in London*
J}ifeafes admitted under the Care of the Phyjicians of the WeJIfninJitr
Ho/pital, from the zoth of May to the z\Jl of June, j8oo.
Continued Fever - - - 16
Pleurify ------ 1
Sore Throat - - - - 1
Amenorphcea - - - -2
Anafarcav ----- 6
Angina Pe&oris - - - - 1
Afcites ------ 2
Afthenia - - - - - - 2
Catarrh ------ 1
Cephalaea - - - - - 1
Cholera ------ 2
Cough ------ 4.
Diarrhoea - - - - - 5
Dyfpepfia - * - - - 1
Dyfuria ------ 2
Enterodynia - * - - 2
Hsemoptyfis - - - - - t
Haemorrhois - --I
Hyfteria ^
Impetigo - - * - - I
Jaundice - -/. ? - - i
Leucorrhoea ----- J
Lithiafis ------ |
Rheumatifm - - ? - 5
Palpitatio ----- 1
Syncope ------ t
Sciatica 1
Struma ------- J
Syphilis ------ 1
Tinea ------ 2
Vertigo ------ 1
Worms I

				

